# Physical exercises to prevent and rehabilitate hospital-associated disability in hospitalised older people: A protocol for a living systematic review with network meta-analysis

**DOI:** 10.12688/f1000research.161912.1

**Published:** 2025-04-08

**Authors:** Katia Giacomino, David Beckwée, Simone C. Gafner, Sophie Wist, Roger Hilfiker, Jan Taeymans, Karl Martin Sattelmayer

**Affiliations:** 1 School of Health Sciences, HES-SO Valais-Wallis, Department of Physiotherapy, Leukerbad, Valais, 3954, Switzerland; 2 Department of Physiotherapy, Human Physiology and Anatomy, Rehabilitation Research (RERE) Research Group, VUB Faculty of Physical Education and Physiotherapy, Brussels, Brussels, Belgium, Belgium; 3 Independent researcher at Physiotherapie Tschopp & Hilfiker, Glis, Valais, 3902, Switzerland; 4 Department of Health Professions, University of Applied Sciences Bern, Bern, Bern, 3005, Switzerland; 5 VUB Faculty of Physical Education and Physiotherapy, Brussels, Brussels, Belgium

**Keywords:** Physical exercise, physical interventions, hospital-associated disability, living systematic review, functional decline, activities of daily living

## Abstract

**Background:**

During hospitalisation, older adults are at risk of developing functional decline

unrelated to the condition for which they were admitted. The loss of independence in at least one activity of daily living is referred to as hospital-associated disability (HAD). A loss of functional independence in hospitalised older people is associated with a greater risk of nursing home placement and increased caregiver burden after hospital dismissal. It is essential to raise awareness of the HAD problem among older patients and to implement adequate preventive and treatment measures.

**Objectives:**

To evaluate systematically the effectiveness of physical exercise to prevent and rehabilitate HAD in people aged 65 years and over who are hospitalised in an acute care setting. To assess factors (training intensity, volume, and frequency) that potentially influence the effectiveness of physical exercise on HAD in such patients through meta-regression.

**Methods:**

We will conduct a living systematic review of randomised controlled trials including a network meta-analysis. For the outcome HAD, various physical exercise modalities such as resistance training, aerobic exercises or cycling could be proposed. Unlike pair-wise meta-analyses, which allow only head-to-head comparisons, network meta-analysis enables us to compare all modalities simultaneously. Guidelines from the Cochrane Handbook for a systematic review of interventions will be followed.

**Discussion:**

A network meta-analysis offers several advantages which are relevant in the context of the present review question: i) it allows the integration of multiple comparisons within one analytical framework; ii) it enables to the evaluation of the comparative effectiveness of each exercise modality, thus allowing for a hierarchy of interventions; iii) it increased precision of the effect estimates compared to traditional meta-analyses. The latter advantage is crucial for outcomes like functional decline after a hospital stay.

## Abbreviation

An overview of used abbreviations is presented in Table 1.

**Table 1.** Abbreviations and its full form.

Abbreviation	Full form
ADL	Activity of daily living
AI	Artificial intelligence
FIDS	Functional Independence and Difficulty Scale
GRADE	Grading of Recommendations Assessment, Development and Evaluation
HAD	Hospital-associated disability
LSR	Living systematic review
NMA	Network meta-analysis
OSF	Open Science Framework
PRISMA	Preferred Reporting Items for Systematic reviews and Meta-Analyses
RCT	Randomised controlled trials
RoB	Risk of bias
SD	Standard deviation
SMAF	Functional autonomy Measurement system
SMD	Standardised mean Difference
WHO	World Health Organisation

## Introduction

During hospitalisation for an acute illness, older adults are at risk of developing functional decline unrelated to the condition for which they were admitted ( Covinsky et al., 2011). Hospital-associated disability (HAD) refers to the loss of independence in at least one activity of daily living (ADL) (e.g., bathing, dressing, etc.) at discharge compared to the individual's ability before hospitalisation ( Covinsky et al., 2011). HAD is not only the disability acquired during a patient’s hospitalisation but also the worsening of a present disability due to hospitalisation ( Covinsky et al., 2011). Dependence in ADLs is often assessed with tools such as the Barthel Index, the Katz Index and the Functional Independence Measure ( Yang et al., 2014).

As the population of older adults increases and hospital admissions rise, understanding ( Loyd et al., 2020; WHO, 2025) and addressing HAD becomes increasingly important.

The number of older people aged 60 years and older will double by 2050 compared to 2025 ( WHO, 2025), it is therefore likely that the number of hospital admissions for the older population will increase, and consequently that the number of people developing HAD will increase as well. According to current reports, the prevalence of HAD is 30% in older adults ( Loyd et al., 2020). A recent systematic review reported a 37% incidence of HAD in older people ( Giacomino et al., 2022). This finding suggests that the problem of functional decline due to hospitalisation is an important issue for relevant stakeholders and should be carefully tackled.

In the hospital environment, patients are inactive most of the time ( Fazio et al., 2020). Hospitalised patients are sitting or lying in bed for approximately 87-100% of the day ( Fazio et al., 2020). Inpatients monitored over 24 hours stood and walked for only 70 minutes per day (95% CI 57 to 83 minutes per day) ( Fazio et al., 2020). In comparison, at home, older people are active during approximately 7.8 hours per day ( Cabanas-Sánchez et al., 2019). This includes about 0.4 hours of moderate to vigorous physical activity, and 7.3 hours of light physical activity ( Cabanas-Sánchez et al., 2019). Within this light physical activity, they spend around 1.6 hours standing and 5.1 hours walking ( Cabanas-Sánchez et al., 2019). A short stay in an acute ward (≤5 days) combined with periods of prolonged immobilisation in bed in the hospital is enough to decrease muscle mass, resulting in changes in muscle strength and muscle function ( Marusic et al., 2021). Hospitalisation in an acute care setting can have negative health consequences for older people, including the loss of functional independence and physical performance ( Valenzuela et al., 2020).

Regular physical activity is recommended in older people. The World Health Organisation (WHO) advises older people to perform at least 150-300 minutes of moderate-intensity or at least 75-150 minutes of aerobic physical activity at vigorous-intensity ( WHO, 2020) per week respectively. To gain additional benefits from training, older adults should incorporate muscle-strengthening exercises two days per week at moderate or higher intensity, as well as engage in multicomponent training (including functional balance and strength) three days per week, also at moderate or greater intensity ( WHO, 2020).

After hospital discharge, patients who have not recovered within one month are at a high risk of developing long-term disability. In fact, only 17.1% regained their baseline function one year later, 44.4% had died, and 38.4% had not recovered ( Boyd et al., 2008). A loss of functional independence in hospitalised older people is associated with a greater risk of nursing home placement ( Brown et al., 2004; Fortinsky et al., 1999) and increased caregiver burden after hospital dismissal ( Covinsky et al., 1997, 1999; Fortinsky et al., 1999).

It is essential to raise awareness of the problem of HAD in clinicians working with hospitalised older patients in order to implement adequate preventive and treatment measures ( Loyd et al., 2020).

### Current state of research in the field

A systematic review 15 studies from 12 RCTs including 2618 patients reported that supervised exercise was effective in improving or attenuating the decline in functional independence and physical performance in older adults hospitalised for acute care ( Valenzuela et al., 2020). More recently, Hartley et al. (2022) conducted a systematic review and evaluated the benefits and harms of exercise interventions in hospitalised older adults on functional ability. The same researchers observed statistically significant effects of exercise interventions on the ability to perform ADLs, in comparison to usual care. However, the differences were not clinically important ( Hartley et al., 2022).

The published systematic reviews are limited, and several questions remain unanswered when it comes to preventing HAD in older patients hospitalised in an acute setting. Hartley et al. (2022) excluded studies investigating management of HAD in the rehabilitation setting ( Parker et al., 2015; Stevens-Lapsley et al., 2016). While it is well-established that HAD can have a long-term impact on ADL disability, previews systematic reviews have not examined the effects of physical interventions for rehabilitating HAD. The previously mentioned systematic reviews ( Bachmann et al., 2010; Hartley et al., 2022; Valenzuela et al., 2020) considered studies with control usual care or a sham-control intervention and these researchers excluded studies with a control group receiving additional exercise. It is important to include such studies in a review to have an overview of the effectiveness of physical exercise interventions in hospitalised older adults in an acute setting. Since the Hartley et al. (2022) study was published ( Hartley et al., 2022), new RCTs have been published ( Carrasco Paniagua et al., 2022; Godino et al., 2022) adding new evidence to the status quo.

A recent network meta-analysis (NMA) investigated the optimal dose and type of physical exercise activity to improve functional capacity and decrease adverse events in acutely hospitalised people over 50 years of age ( Gallardo-Gómez et al., 2023). Our project differs from theirs in several respects: i) our study will include articles with older population (over 65 years of age), which does represent the typical geriatric population, ii) our study will investigate a broad range of types of physical exercise interventions, iii) the long-term effects on functional capacity will be studied and iv) our NMA will be based on a living systematic review (LSR) which will be updated over three years.

We plan to carry out an NMA because it offers several advantages which are relevant in the context of the present review questions: i) NMA allows the integration of multiple comparisons within one analytical framework. For the outcome HAD various physical exercise modalities such as resistance training, aerobic exercises or cycling have been proposed. Unlike pair-wise (head-to-head) meta-analyses (e.g. resistance training vs. aerobic exercises), NMA enables us to compare all modalities simultaneously; ii) NMA enables us to assess the comparative effectiveness of each exercise modality, thus allowing us to propose a hierarchy of physical exercise interventions; iii) NMA increases precision of the effect estimates compared to traditional meta-analyses ( Chaimani et al., 2019). The latter advantage of NMA is crucial for outcomes like functional decline in older patients after a hospital stay.

### Objectives

In the first year a systematic review on the effectiveness of physical exercise intervention to prevent and rehabilitate HAD in patients aged 65 years and more hospitalised in acute setting will be performed. In the consecutive three following years the systematic review will regularly be updated using a LSR approach.

We will also assess factors (training intensity, volume, and frequency) that potentially influence the treatment effectiveness on HAD in such patients through meta-regression.

Regarding the distinction between the prevention and rehabilitation we will use the following classification: depending on the setting of the interventions we will analyse the effect of preventive physical exercise on HAD in older, hospitalised patients (i.e. the exercises are performed within the acute care setting in order to minimise a potential HAD). If physical exercise interventions are administered in a rehabilitation setting after a hospital stay, we will analyse the effect of rehabilitation on HAD (i.e. the exercises are used to reduce HAD).

The primary outcome of this review will be short term basic functional ability in performing ADL tasks following hospitalisation discharge or rehabilitation discharge (between 0 and 14 days after hospital or rehabilitation discharge).

Secondary outcomes will include i) long term basic functional ability in performing ADL tasks in older patients following hospitalisation or rehabilitation discharge (at least 15 days after discharge). Furthermore, we will analyse the following secondary outcomes: ii) balance; iii) strength; iv) endurance; v) walking abilities; vi) length of stay, vii) re-hospitalisation rate; viii) adverse events and ix) mortality rate.

We will compare the active interventions (i.e. different types of physical exercise) whit each other in the NMA but we will also include non-active comparators such as usual care or sham interventions into the analysis.

## Methods

### Detailed research plan

We will conduct a living systematic review of RCTs with network meta-analysis. Guidelines from the Cochrane Handbook for a systematic review of interventions will be followed ( Higgins et al., 2023).

### Eligibility criteria

*Types of studies that will be included*

We will include only published RCTs and cross-over studies if they fulfil the condition of randomised allocation of study participants. Other designs, such as non-randomised clinical trials, retrospective studies, cohort studies, single case reports and letters to editors, will be excluded. Articles in English, French, German, and Italian will be included. Other languages will be excluded if the automatic translation with applications such as Google translate ( https://translate.google.com/) or Deepl ( https://www.deepl.com/) is not sufficient to understand the content of the study. No restrictions will be placed on the date of publication.

*Types of participants and condition to be studied*

We will include studies that investigated older adults, aged 65 years and above, who were hospitalised in acute care setting (e.g. hospitalised for an acute illness that responds to usual medical management ( Covinsky et al., 2011)) and at risk of developing HAD. HAD refers to disabilities acquired during hospitalisation or the worsening of a pre-existing disability due to hospitalisation ( Covinsky et al., 2011). Moreover, we will also include studies investigating older people discharged from rehabilitation centre (after a hospital stay) with a reduced ability to perform ADLs, which were likely caused by hospitalisation. All acute pathologies will be included, except those with a high risk of permanent loss of function, function (e.g., stroke, post COVID-19 condition, fractures, cancer), which are directly disabling.

*Types of interventions*

In this project, we will include all studies that have assessed physical exercise interventions targeting the prevention and rehabilitation of HAD in older, hospitalised patients. Combined exercises will also be eligible (e.g., resistance training and balance training). Reports analysing exercise programmes including strengthening and mobility exercises compared to usual care in such patients will also be eligible ( Jones et al., 2006). Studies implementing cognitive exercise without physical exercises to prevent or rehabilitate HAD in such patients will be excluded. For example, studies investigating cognitive training in fully immersive virtual reality compared to pharmacology to prevent or rehabilitate HAD in older, hospitalised patients will be excluded ( Kang et al., 2021).

*Types of comparators*

Studies included in this systematic review with NMA must investigate at least two groups and one of them must be active (i.e., performing physical exercises). The control group can be a non-active intervention (such as a control or sham intervention).

*Types of outcomes*

We will include studies that evaluated the immediate and long-term effectiveness of physical exercise on the ability to perform basic ADLs in older patients with HAD following acute hospital care. Additionally, we will consider studies that investigated the effectiveness of these interventions on balance, strength, endurance, walking abilities, length of hospital stays, rehospitalisation rate, adverse events and mortality rates.

### Information sources

We will perform the literature search on four electronic bibliographic databases: PubMed (MEDLINE), Embase (via Embase.com), CENTRAL (Cochrane Central Register of Controlled Trials, The Cochrane Library) and CINAHL Ultimate (via EBSCO). The literature search algorithm will first be developed for Medline (PubMed) and then adapted for implementation on the other databases.

All four electronic databases will be screened from inception until present. The search algorithm will be updated every two months for a period of three years from August 2025 on.

We will use the recommended highly sensitive filters for clinical trials suggested by the Cochrane Collaboration ( Higgins et al., 2023). Grey literature will be searched by consulting Google Scholar (RRID: SCR_008878), the reference list of important publications on the topic, WHO International Clinical Trials Registry Platform (RRID: SCR_004475), and ClinicalTrials.gov (RRID: SCR_002309). Articles will be searched from inception to the present.

### Search strategy

The search strategy will be developed using the litsearchR package ( Grames et al., 2019). The following steps will be performed: First, we will perform a naïve search on PubMed using the search terms “hospitalised older people”, “physical exercise” and “functional decline”. The identified records will be downloaded. We will extract potentially relevant search terms from title, abstract and keywords section using Rapid Automatic Keyword Extraction ( Rose et al., 2010). In a follow up-step a term and keyword co-occurrence network will be established. The parameters will be set as follows: i) a term had to occur at least in three studies to be included; ii) the minimum total number of times a term have to occur will set to three. We used the ggraph package ( Pedersen, 2023) to visualise the network. To identify the most relevant terms we will rank the terms with regard to their strength in the network (i.e. the number of times they co-occur with other terms). We will set the cut-off point for inclusion of search terms to 80% strength and remove the remaining terms. The identified terms will be classified into four search concepts (i.e. terms related to: i) population such as older people; ii) setting (i.e. acute hospital); iii) interventions such as physical exercise and iv) outcome such as functional decline. Terms within each concept will be combined using the Boolean operator “OR”. All four concepts will be combined using “AND”. The search string will be further refined on the individual database such as adding specific subject headings and terms which will not pass the threshold of but will be deemed relevant by the review team. In addition, we will add the database specific methodological filter for clinical trials to the search string ( Higgins et al., 2023).

To validate the search string we will identify six studies, which fulfil our selection criteria ( Blanc-Bisson et al., 2008; Brown et al., 2016; Hu et al., 2020; Martínez-Velilla et al., 2019; McCullagh et al., 2020; Ortiz-Alonso et al., 2020). We will select the reference standard from the systematic reviews of Valenzuela (2020) and Gallardo-Gómez et al. (2023). A search on Embase and PubMed will be identified all studies of our reference standard. The McCullagh study (2020) is not indexed in Embase but can be identified with the PubMed search. The search string for PubMed is presented in Table 2.

**Table 2.** Four search concepts combined with the Boolean operator “AND.

**Search concept population**	(“65 year” [All Fields] OR “aged” [MeSH Terms] OR “ageing” [All Fields] OR “aging” [MeSH Terms] OR “community-dwelling older” [All Fields] OR “community-dwelling older adults” [All Fields] OR “elderly” [All Fields] OR “frail older” [All Fields] OR “frailty” [All Fields] OR “geriatric patients” [All Fields] OR “healthy older” [All Fields] OR “healthy older adults” [All Fields] OR “older adult” [All Fields] OR “older adults” [All Fields] OR “older medical patients” [All Fields] OR “older patient” [All Fields] OR “older patients” [All Fields] OR “older people” [All Fields] OR “older population” [All Fields] OR “pre-frail” [All Fields])
**Search concept setting**	(“acute care” [All Fields] OR “acute hospitalisation” [All Fields] OR “acute hospitalization” [All Fields] OR “acute medical” [All Fields] OR “acutely hospitalised” [All Fields] OR “acutely hospitalised older” [All Fields] OR “acutely hospitalized” [All Fields] OR “acutely hospitalized older” [All Fields] OR “Hospitalisation-associated disability” [All Fields] OR “hospital admission” [All Fields] OR “hospital discharge” [All Fields] OR “hospitalisation” [All Fields] OR “hospitalization” [All Fields] OR “hospitalised” [All Fields] OR “hospitalized” [All Fields] OR hospitals [MeSH Terms] OR “physical rehabilitation” [All Fields] OR “Posthospitalisation” [All Fields] OR “Posthospitalization” [All Fields] OR “primary care” [All Fields] OR “public hospital” [All Fields] OR “rehabilitation centre” [All Fields] OR “rehabilitation centers” [MeSH Terms] OR “rehabilitation clinic” [All Fields] OR “tertiary public” [All Fields] OR “tertiary public hospital” [All Fields])
**Search concept intervention**	(“aerobic exercise” [All Fields] OR “activity program” [All Fields] OR “activity programme” [All Fields] OR “exercise” [MeSH Terms] OR “exercise group” [All Fields] OR “exercise intervention” [All Fields] OR “exercise program” [All Fields] OR “exercise programme” [All Fields] OR “exercise programs” [All Fields] OR “exercise programmes” [All Fields] OR “exercise sessions” [All Fields] OR “exercise training” [All Fields] OR “Gamification” [All Fields] OR “game-based” [All Fields] OR “physical activity” [All Fields] OR “physical activity program” [All Fields] OR “physical activity programme” [All Fields] OR “physical exercise” [All Fields] OR “physical exercise program” [All Fields] OR “physical exercise programme” [All Fields] OR “physical performance” [All Fields] OR “physical therapy” [All Fields] OR “Physiotherapy” [All Fields] OR “programme performed” [All Fields] OR “program performed” [All Fields] OR “progressive resistance” [All Fields] OR “reablement program” [All Fields] OR “reablement programme” [All Fields] OR “resistance exercise” [All Fields] OR “resistance training” [All Fields] OR “resistance training” [MeSH Terms] OR “tailored exercise” [All Fields] OR “tailored exercise program” [All Fields] OR “tailored exercise programme” [All Fields] OR “training program” [All Fields] OR “training programme” [All Fields])
**Search concept outcome**	(“ADL” [All Fields] OR “activities of daily living” [All Fields] OR “activities of daily living” [MeSH Terms] OR “autonomy” [All Fields] OR “balance” [All Fields] OR “barthel index” [All Fields] OR “battery score” [All Fields] OR “daily living” [All Fields] OR “frailty score” [All Fields] OR “functional ability” [All Fields] OR “functional capacity” [All Fields] OR “functional decline” [All Fields] OR “functional performance” [All Fields] OR “functional status” [All Fields] OR “health-related quality” [All Fields] OR “independent living” [All Fields] OR “instrumental activities” [All Fields] OR “Katz” [All Fields] OR “mobility” [All Fields] OR “mobility impairment” [All Fields] OR “performance battery” [All Fields] OR “performance battery score” [All Fields] OR “physical decline” [All Fields] OR “physical function” [All Fields] OR “physical functioning” [All Fields] OR “physical performance” [All Fields] OR “short physical” [All Fields] OR “skeletal muscle” [All Fields] OR “strength” [All Fields])
**Search concept methodological filter**	(“randomized controlled trial” [pt] OR “controlled clinical trial” [pt] OR randomized [tiab] OR placebo [tiab] OR drug therapy [sh] OR randomly [tiab] OR trial [tiab] OR groups [tiab] NOT (“animals” [MeSH Terms] NOT humans [MeSH Terms]))

### Study records

*Data management*

Bibliographic data will be downloaded from the databases and stored on a secured cloud platform with servers located in Switzerland. We will use the AsysD package ( Hair et al., 2023) to de-duplicate records. The Covidence tool ( Covidence systematic review software) will be used for screening and data extraction.

*Selection process*

For the first screening phase, two human reviewers (SW, KG, MS or SG) will independently screen all references by reading the titles and abstracts. In the pilot phase, in parallel with the evaluation of the human evaluators, a selection of articles by artificial intelligence (AI) will also be carried out. The results of the relevant articles included by the two human reviewers supported by AI-software (such as https://asreview.nl/) will be compared. In case of different results, another human reviewer (SG or MS) will be involved in the decision-making process and the two people will find a consensus through discussion. If no consensus is found, the article will automatically be included in the full-text reading. In the living systematic review study phase, if the results of the articles selection obtained by the AI-software match the results of the human assessors, we will be making the selection using only a human reviewer supported by AI.

At the full-text selection stage, articles will be independently reviewed by two human (SW, KG or MS) reviewers according to the inclusion criteria. If there is any disagreement, a third person (SG) will make the final decision. Articles included in the systematic review will be incorporated into the NMA if they provide sufficient information about outcome data. If data is missing, we will reach out to the original authors for further information.

*Data collection process*

One reviewer will extract data manually (SW or MS) and a second reviewer (KG or SG) will control them independently. Disagreements and errors will be discussed and resolved through a joint agreement. Data extraction will be performed though forms designed in Power App (RRID: SCR_021865) and the functionality of the forms will be tested by on person (SW) by integrating different studies.

*Data items*

The following information will be extracted for the study characteristics: authors of the publication, year of publication, country of study, age of participants, number of participants randomised, gender proportion, pathologies, type of physical exercise, training modalities (frequency of interventions, duration, and intensities), type of control intervention, length of intervention. For the following variables under investigation, we will extract the central tendencies and measures of variation: score of functional ability assessment tools (e.g. Katz Index, Barthel Index, etc.), physical performance parameters (e.g. balance, strength, endurance, and walking abilities). We will also extract hospital length of stay, the rehospitalisation rate, adverse events and mortality rate.

### Outcomes and prioritisation

*Primary outcomes*

The short-term effectiveness of physical exercise interventions that target prevention of HAD and the rehabilitation of HAD in older hospitalised patients will be assessed in the following ways:

• Short-term basic functional ability at the end of hospitalisation (i.e., discharge from acute care setting). We will recognise the period from 1 to 14 days after discharge from the hospital as belonging to the disability due to hospitalisation.
• Short-term basic functional ability at the end of rehabilitation interventions (i.e., discharge from rehabilitation setting). We will recognise the period from 1 to 14 days after discharge from the rehabilitation setting as belonging to the disability due to hospitalisation after a rehabilitation period.

All tools designed to measure basic ability in everyday activities will be considered for inclusion. Examples of potential relevant tools are the Barthel Index ( Mahoney & Barthel, 1965), the Functional Independence Measure ( Keitll et al., 1987) or the 5-items Katz Index ( 1963).

If a study uses different measures of ADLs, the order of priority for extraction of the measures is based on the recommendations of Hopman-Rock et al. (2019): the Functional Autonomy Measurement System (SMAF) ( Boissy et al., 2007), 5-items Katz Index ( 1963), Functional Independence and Difficulty Scale (FIDS) ( Saito et al., 2016), and the Barthel Index ( Mahoney & Barthel, 1965).

*Secondary outcomes*

The long-term effect of physical exercise interventions (i.e. for prevention and rehabilitation of HAD in older, hospitalised patients in an acute care setting) on ADLs will be investigated as secondary outcome. ADLs will be assessed at the last available time point, provided that other follow-up measures are available. We have defined long-term disability after hospital discharge with a period of interest from 15 to 90 days after hospital discharge.

• Long-term basic functional ability after hospital discharge. We will recognise the period from 15 to 90 days after discharge from the hospital as belonging the long-term disability due to hospitalisation.
• Long-term basic functional ability after the rehabilitation programme. We will recognise the period from 15 to 90 days after discharge from rehabilitation setting as belonging to the long-term disability due to hospitalisation.

Additional outcomes will include the investigation of other health parameters affected by physical inactivity due to bed rest such as balance, strength, endurance, and walking abilities will be assessed. We will also assess length of stays, rehospitalisation rate, adverse events and mortality rate.

*Risk of bias in individual studies*

For risk of bias (RoB) assessment, we will use the Cochrane Group's RoB 2.0 tool ( Sterne et al., 2019). RoB 2.0 will be evaluated independently by two reviewers (SW, KG or SG). The differences in evaluation will be discussed between the two reviewers until a consensus is reached. RoB will be analysed for the primary outcome, i.e. dependence on ADLs. In a sensitive analysis, we will remove articles that have been identified as having a high RoB. A high risk of bias refers to situations where there are serious concerns about the methodological quality of the study that are likely to lead to misleading or unreliable results. The RoB 2.0 (Risk of Bias 2.0) tool, assess five domains: i) bias arising from the randomisation process; ii) bias due to deviations from the intended interventions; iii) bias due to missing outcome data; iv) bias in measurement of the outcome; v) bias in selection of the reported result ( Sterne et al., 2019).

A low risk of bias is considered when all five domains of the RoB 2.0 are rated with minimal to no concerns that the results could be affected by systematic errors ( Sterne et al., 2019).

### Data synthesis

*Pair-wise meta-analyses*

The statistical analyses will be performed using the statistical software package R ( Team, 2023). In the first step, we will use the “meta” package ( Schwarzer et al., 2015) to analyse the direct evidence with pair-wise meta-analyses (i.e., the same interventions will be analysed together). For example, all available trials reporting aerobic exercise versus a control intervention will be analysed together. This “head-to-head” approach will be repeated for all identified comparisons and will provide direct evidence of the effectiveness of the physical exercise interventions on HAD in older adults. A head-to-head comparison in meta-analyses is defined as the direct comparison of two interventions in the same study. This type of comparison is straightforward because it is based on the analysis of data in which the interventions have been directly compared against each other in RCTs.

*Summary measures for pair-wise meta-analyses*

For the analysis of continuous outcomes, number of study participants, means and standard deviations (SDs) of the included studies will be used. If the study means are expressed in the same units (e.g., all studies use the Barthel Index), we will use the between-group raw mean difference as effect size. In cases where the mean is expressed in different units across studies, the standardised between-group mean difference (SMD) will be selected as the effect size ( Takeshima et al., 2014). SMD will be interpreted according to Cohen (1988). This means that an effect size around 0.2 will be considered as small, 0.5 as medium and 0.8 as large. Dichotomous outcomes will be analysed using Peto odds ratios ( Yusuf et al., 1985). We will employ the Peto method for effect size calculation. The between study variance (Tau ^2^) will be estimated using the Paule-Mandel estimator.

*Method of analysis for pair-wise meta-analyses*

As we assume that the included studies have some differences in terms of interventions and study populations, a random-effects model will be used for the analyses. The Sidik-Jonkman approach will be used to determine the between-study heterogeneity ( Sidik & Jonkman, 2005). In addition, the Hartung-Knapp adjustment for confidence intervals will be used ( Hartung, 1999; Hartung & Knapp, 2001).

*Assessment of inconsistency for pair-wise meta-analyses*

We will assess statistical heterogeneity using the Chi-squared test with its corresponding degrees of freedom and p-value. Higgins’ I ^2^ statistic will be calculated as a measure of heterogeneity following the Cochrane Handbook recommendations ( Higgins et al., 2023). In addition, we will calculate and use tau-squared to quantify the variance of the true effect sizes and tau for the standard deviation of the true effect sizes ( Borenstein et al., 2021).

*Analysis of moderator variables for pair-wise meta-analyses*

The second step will be to perform a meta-regression in R using the “meta” package ( Schwarzer et al., 2015). A mixed effects model will be used, and a restricted maximum likelihood estimator will be employed for estimating between-study variance (Tau ^2^). We will explore the effect of the following potential moderator variables on the estimated effect of the HAD interventions: i) intervention volume (the total dose of received intervention in minutes); ii) average hospitalisation duration and iii) average age of the study participants. According to Paterson and Warburton (2010), there seems to be a dose-response relationship between physical activity and functional outcomes. However, the adequate treatment ‘dose’ in older adults in terms of recommendation is unclear. There is evidence that ‘higher’ levels of physical activity are advantageous to be independent in ADL. Therefore, it is necessary to establish if there is a minimum threshold of physical activity in order to achieve benefits in functional independence in older adults ( Paterson & Warburton, 2010).

*Network meta-analysis*

In a second step a frequentist NMA will be performed using the “netmeta” package of the R software ( Rücker et al., 2015). The transitivity assumption will be examined for all studies included in the NMA ( Salanti et al., 2014). Firstly, we will investigate whether potential effect modifiers are sufficiently similar between studies. For this, we will investigate important clinical and methodological characteristics such as the severity of illness at baseline (e.g. proportion of participants hospitalised for acute conditions, the pathologies, the age of participants and reasons for admission), sample size and study quality (or RoB). This evaluation will be based on a clinical understanding of the disease (i.e. the research team will review this characteristics). If sufficient studies are available, we will statistically assess the distribution of the effect modifiers ( Chaimani et al., 2017). Secondly, we will check whether all interventions are meaningful to all participants. This means that any participant could theoretically receive any treatment in the network of interventions and that the included trials must be sufficiently similar, except for the treatments being compared ( Chaimani et al., 2017). If the requirement for a NMA is not met, we will analyse results with a standard pair-wise meta-analysis.

*Geometry of the network*

The network of treatments will be visualised using network plots using the “netmeta” package of the R software ( Rücker et al., 2015). Each intervention will be represented as a circle (i.e. the network nodes) and circles will be connected with lines (i.e. edges) if a direct comparison (i.e. head-to-head comparison) between two interventions is available. The network will be examined for the presence of open and closed loops. A closed loop refers to comparisons with multiple pathways, incorporating both direct and indirect evidence. An open loop refers to comparisons lacking direct evidence ( Antoniou et al., 2019). To facilitate interpretation of the network the comparisons will be colour coded.

*Summary measures for NMA*

We will use the same summary measures in the NMA as proposed for the pair-wise meta-analyses above. That is, standardised mean differences will be used for continuous outcomes and Peto odds ratios ( Yusuf et al., 1985) will be used for dichotomous outcomes. A treatment ranking will be produced using the P-score, which is the frequentist equivalent to the SUCRA score ( Rücker & Schwarzer, 2015). The SUCRA score (is a statistical measure employed in network meta-analysis to rank treatments based on their effectiveness. It provides a single number that reflects the likelihood that a treatment is the best option among all alternatives. A higher SUCRA score indicates a higher likelihood of the treatment being the most effective ( Rücker & Schwarzer, 2015).

*Planned method of analysis for NMA*

Assuming a relatively high degree of clinical and methodological heterogeneity between the included studies, we will perform a random-effects model. As treatment effects in multi-arm studies are not independent, comparisons of each multi-arm study will be reweighted following Rücker and Schwarzer (2014).

*Assessment of inconsistency*

Heterogeneity and inconsistency will be quantified with Higgins’ I ^2^ statistics ( Higgins et al., 2019) using the “netmeta” package of the R software ( Rücker et al., 2015). The value of heterogeneity and inconsistency will be interpreted in accordance with the Cochrane Handbook: 0% to 40% may be considered as not important; 30% to 60% may represent moderate heterogeneity; 50% to 90% may represent substantial heterogeneity; 75% to 100% considerable heterogeneity ( Higgins et al., 2019). In addition, heterogeneity (within designs) and inconsistency (between designs) will be explored. A comparison of direct treatment estimates and indirect estimates will be performed to check the robustness of the indirect treatment estimates. This comparison will be supported by i) net league tables contrasting direct effect estimates and network effect estimates and ii) net split forest plots showing direct, indirect and network effect estimates. Furthermore, we will visualise inconsistency with net heat plots.

*Additional analyses*

Planned separated sensitivity analyses will be performed by including only studies with an overall low RoB rating (i.e. the network metanalysis will be performed with the same parameters described above and considering only studies with a low overall RoB rating), or on specific domains of the RoB tool 2.0 ( Sterne et al., 2019) (randomisation process, deviation from intended interventions, missing outcome data, measurement of the outcome, and selection of reported results). Additional post hoc sensitivity analyses will be considered to evaluate if the results are robust to the choices that were made during the review process such as analysis of cross-over studies or imputed standard deviation values. Missing values such as means and standard deviation will be imputed following the Cochrane Handbook (ref ) and if we are in doubt about an imputed value sensitivity analyses will be performed.

*Meta-bias(es)*

We will use comparison adjusted funnel plots to investigate a possible publication bias ( Chaimani & Salanti, 2012) using the “netmeta” package of the R software ( Rücker et al., 2015). In order to use this statistical approach, we will check if the studies can be ordered in a meaningful way after inclusion of all studies ( Salanti et al., 2014). A possible order could be the degree of intensity ranging from low intensity exercises to high intensity exercises. Other possible order variables could be: i) the novelty of the exercise type ranging from traditional exercise over gamified exercises to virtual reality supported exercises; or ii) meaningful functional exercises vs analytical exercises.

*Grading of the level of evidence*

We will use the Confidence in NMA (CINeMA, RRID: SCR_023193) framework ( Salanti et al., 2014) ( https://cinema.ispm.unibe.ch), which is based on the Grading of Recommendations Assessment, Development and Evaluation (GRADE), to rate the quality level of the evidence. If a NMA cannot be performed, we will use the standard grading system from the GRADE working group ( Schünemann et al., 2013).

## Discussion

We will conduct a LSR with NMA on physical exercise interventions that target (prevention and rehabilitation) HAD in older, hospitalised adults (aged 65 years and over). A key reason for selecting a LSR design is that the results of this method will allow us to create an exercise intervention that will be continuously updated based on the latest evidence in the literature. This approach aims both to prevent the decline in the ability to perform ADLs in individuals at risk of developing HAD and to rehabilitate patients who have developed HAD and require rehabilitation. For example, the initial analysis might find that resistance training is the most effective for preventing HAD in such patients. We would then design a training program around resistance exercises. However, if future updates indicate that another exercise, such as long-distance walking, is more effective, we will adapt the program accordingly. To our knowledge, this is the first LSR directly linked to an adaptive health intervention.

The LSR approach consists in continually running the searches and rapidly incorporating the identified new evidence in the results. The LSR approach is relatively new and is defined as a continuously updated systematic review, incorporating new relevant data as they become available. The advantages of a LSR are: i) the time before the new analysis will be conducted will be shortened, which will benefit clinicians and readers; ii) readers have access to the latest version of our project results, allowing for direct correction and updating of any errors, including those in analysis. In practice, corrections are seldom made. When they are, there is a significant RoB if only the original paper is considered; and iii) clinicians will be able to rapidly integrate new evidence into practice, thereby enhancing the quality of care for older adults.

### Dissemination

Once the initial systematic review is completed, the article will be published in an open access peer-reviewed journal. Afterwards the bi-monthly search update of the results will be presented on the project website ( https://had-prevention.netlify.app). In case of relevant changes of the findings an update of the LSR will be submitted to a peer-reviewed journal.

### Study status

Adapting the search string in the different databases.

#### Ethical considerations

Ethical approval and consent were not required.

## Data Availability

No data are associated with this article. OSF. Title of the project: Physical exercises to prevent and rehabilitate hospital-associated disability in hospitalised older people: A living systematic review with network-meta-analysis ( Giacomino et al., 2025). The project was registered on OSF October 7, 2024. At the end of the project, the data and metadata generated during the project will be stored on the Open Science Framework (OSF) server. Data are available under the terms of the Creative Commons Attribution 4.0 International license (CC-BY 4.0). The Preferred Reporting Items for Systematic reviews and Meta-Analyses (PRISMA, RRID: SCR_018721) statement extension of NMA will be used for reporting ( Hutton et al., 2016). This protocol follows the recommendations of the PRISMA guideline ( Moher et al., 2015). The document can be downloaded through the following link: https://osf.io/8fjtd/files/osfstorage.
