# Supplementary material for: Physical exercises to prevent and rehabilitate hospital-associated disability in hospitalised older people: A protocol for a living systematic review with network meta-analysis
Source: F1000Res. 2025 Apr 8;14:414. [Version 1] doi: 10.12688/f1000research.161912.1 (PMC12541298; doi:10.12688/f1000research.161912.1)
Supplement: Peer review history

# Reviewer report: Reviewer response for version 1

**DOI:** 10.5256/f1000research.178009.r419798

**Published:** 2025-10-21

**Authors:** Christi Deaton

**Affiliations:** 1 University of Cambridge School of Clinical Medicine, Cambridge, UK

This is an interesting protocol for a living systematic review that has the potential for great benefit to researchers and practitioners. My main concern is around combining analysis of interventions within an acute hospitalisation with interventions within a rehabilitation setting.  This needs to be clarified and justified if these are analysed together. During acute hospitalisation, the focus is on treating the underlying illness as well as preventing HAD. This is different from interventions for those who have recovered enough for hospital discharge and the sole focus is now on improvement in HAD/function.  Patients would be in different states as would time for these interventions (often interrupted in acute hospital due to tests, procedures, patient status). 

 Can you clarify the number of articles that will be evaluated by AI during the pilot phase?

 Otherwise the protocol is well described and is using innovative techniques.

 A few typos remain and should be corrected.

Is the study design appropriate for the research question?

Yes

Is the rationale for, and objectives of, the study clearly described?

Partly

Are sufficient details of the methods provided to allow replication by others?

Yes

Are the datasets clearly presented in a useable and accessible format?

Not applicable

Reviewer Expertise:

cardiovascular conditions, especially heart failure,  and interventions including rehabilitation.

I confirm that I have read this submission and believe that I have an appropriate level of expertise to confirm that it is of an acceptable scientific standard.

# Reviewer report: Reviewer response for version 1

**DOI:** 10.5256/f1000research.178009.r382100

**Published:** 2025-06-03

**Authors:** Richard E Kennedy

**Affiliations:** 1 University of Alabama at Birmingham, Birmingham, USA

This manuscript describes the protocol for a planned living systematic review (LSR) of the effectiveness of physical exercise interventions for the prevention and rehabilitation of hospital-associated disability (HAD).  HAD, or decline in activities of daily living, is a significant problem with the aging of the population, and the rates of HAD have not shown any significant reduction over the last 30 years despite the increased recognition of the condition.  Physical exercise remains a promising intervention for HAD, and a comprehensive assessment of its effectiveness will help guide clinicians in recommendations for both prevention and rehabilitation.  The protocol for the review is described in detail, and the methods for identification of relevant articles is well described.  The process of data synthesis is comprehensive and well suited to address the hypotheses, and the statistical analysis is appropriate with several mitigation strategies for potential problems and sensitivity analyses to ensure the robustness of the results.

 There are only a few problems that need to be addressed with the manuscript, two moderate and two minor:

 1) Under outcomes on p. 9, the authors should explicitly address how individuals discharged from hospital to rehabilitation will be handled.  If the 1 to 14 days after hospitalization is part of a rehabilitation stay, will it be counted?  Would the potential effects of the beginning of the rehabilitation stay on the outcome measures be considered?  What about hospitalization followed by a rehabilitation stay of less than 14 days (which is common at least in some countries such as the US) - will this count towards the hospitalization assessment, as well as towards rehabilitation assessment?

 2) Under moderator variables on p. 10, the authors note a dose-response as well as a potential threshold effect of exercise in preventing / rehabilitating HAD, but appear to only describe analyses for the former.  How will threshold effects be incorporated into their analysis?  It seems fairly reasonable that there would be a minimal level of exercise needed to achieve benefit, and that levels beyond that would not afford much added benefit, so a threshold analysis is definitely encouraged.

 3) In the introduction on p. 5, the authors note "our study will include articles with older population (over 65 years of age), which does represent the typical geriatric population", which seems a bit confusing - adults over 65 would actually seem to be the typical geriatric population.

 4) In the secondary outcomes on p. 9, length of stay (LOS) needs special consideration, as it can be both a cause and a consequence of HAD: individuals may have longer LOS to address debilitation due to HAD, but longer length of stay may also be associated with longer periods of immobility and greater likelihood of developing HAD.  Need to address how this will be handled in the analysis, or if it will simply be noted as a limitation.

Is the study design appropriate for the research question?

Yes

Is the rationale for, and objectives of, the study clearly described?

Yes

Are sufficient details of the methods provided to allow replication by others?

Yes

Are the datasets clearly presented in a useable and accessible format?

Not applicable

Reviewer Expertise:

delirium, dementia, and community mobility

I confirm that I have read this submission and believe that I have an appropriate level of expertise to confirm that it is of an acceptable scientific standard.
